# Comparison of molecular quantification of *Plasmodium falciparum* gametocytes by *Pfs25* qRT-PCR and QT-NASBA in relation to mosquito infectivity

**DOI:** 10.1186/s12936-016-1584-z

**Published:** 2016-11-08

**Authors:** Helmi Pett, Bronner P. Gonçalves, Alassane Dicko, Issa Nébié, Alfred B. Tiono, Kjerstin Lanke, John Bradley, Ingrid Chen, Halimatou Diawara, Almahamoudou Mahamar, Harouna M. Soumare, Sekou F. Traore, Ibrahima Baber, Sodiomon B. Sirima, Robert Sauerwein, Joelle Brown, Roly Gosling, Ingrid Felger, Chris Drakeley, Teun Bousema

**Affiliations:** 1 Department of Medical Microbiology, Radboud University Medical Center, Geert Grooteplein Zuid 26-28, 6500 HB Nijmegen, The Netherlands; 2 Department of Immunology and Infection, London School of Hygiene & Tropical Medicine, London, UK; 3 Malaria Research and Training Centre, Faculty of Pharmacy and Faculty of Medicine and Dentistry, University of Science, Techniques and Technologies of Bamako, Bamako, Mali; 4 Centre National de Recherche et de Formation sur le Paludisme, Ouagadougou, Burkina Faso; 5 MRC Tropical Epidemiology Group, Department of Infectious Disease Epidemiology, London School of Hygiene & Tropical Medicine, London, UK; 6 Department of Epidemiology and Biostatistics, University of California San Francisco, San Francisco, CA USA; 7 Molecular Diagnostics Unit, Swiss Tropical and Public Health Institute, Basel, Switzerland

**Keywords:** Malaria, Gametocytes, Quantification, *Anopheles*, Mosquito, QT-NASBA, qRT-PCR, Transmission

## Abstract

**Background:**

Quantifying gametocyte densities in natural malaria infections is important to estimate malaria transmission potential. Two molecular methods (*Pfs25* mRNA quantitative reverse transcriptase PCR (qRT-PCR) and *Pfs25* mRNA quantitative nucleic acid sequence based amplification (QT-NASBA)) are commonly used to determine gametocyte densities in clinical and epidemiological studies and allow gametocyte detection at densities below the microscopic threshold for detection. Here, reproducibility of these measurements and the association between estimated gametocyte densities and mosquito infection rates were compared.

**Methods:**

To quantify intra- and inter-assay variation of QT-NASBA and qRT-PCR, a series of experiments was performed using culture-derived mature *Plasmodium falciparum* gametocytes from three different parasite isolates (NF54, NF135, NF166). *Pfs25* mRNA levels were also determined in samples from clinical trials in Mali and Burkina Faso using both methods. Agreement between the two methods and association with mosquito infection rates in membrane feeding assays were assessed.

**Results:**

Intra- and inter-assay variability was larger in QT-NASBA compared to qRT-PCR, particularly at low gametocyte densities (< 1 gametocyte per μL). Logistic models, including log-transformed gametocytaemia estimated by QT-NASBA, explained variability in mosquito feeding experiment results as well as log-transformed gametocytaemia by qRT-PCR (marginal R^2^ 0.28 and 0.22, respectively). Densities determined by both methods strongly correlated with mosquito infection rates [Spearman’s rank correlation coefficient, 0.59 for qRT-PCR and 0.64 for QT-NASBA (P < 0.001 for both)]. Gametocyte densities estimated by qRT-PCR were higher than levels estimated by QT-NASBA or light microscopy at high densities (>100 gametocyte per μL). Samples collected in one of the two transmission studies had extremely low gametocyte densities by both molecular methods, which is suggestive of RNA degradation due to an unknown number of freeze–thaw cycles and illustrates the reliance of molecular gametocyte diagnostics on a reliable cold-chain.

**Conclusions:**

The experiments indicate that both qRT-PCR and QT-NASBA are of value for quantifying mature gametocytes in samples collected in field studies. For both assays, estimated gametocyte densities correlated well with mosquito infection rates. QT-NASBA is less reproducible than qRT-PCR, particularly for low gametocyte densities.

**Electronic supplementary material:**

The online version of this article (doi:10.1186/s12936-016-1584-z) contains supplementary material, which is available to authorized users.

## Background

The transmission of malaria from humans to mosquitoes requires the presence of mature sexual stage parasites (gametocytes) in the peripheral blood. Upon ingestion by *Anopheles* mosquitoes and following sporogonic development, these gametocytes can render mosquitoes infectious to humans. Quantifying the density of these gametocytes in the peripheral blood is a fundamental part of estimating the infectiousness of individuals with malaria infections. For over a century, light microscopy was the only method available to quantify malaria parasites, including gametocytes. The development of sensitive molecular methods has uncovered some of the limitations of microscopy. In endemic areas, a substantial proportion of infected individuals carry parasites at levels below the microscopic threshold of detection [1–4], and many of these individuals also have low, sub-microscopic, densities of gametocytes [5, 6]. Together with early observations that individuals with no gametocytes detected by microscopy could be infectious to *Anopheles* mosquitoes [7], these studies suggest that the use of more sensitive methods is necessary to characterize the infectious reservoir of malaria [8, 9].

The most widely used target for molecular gametocyte detection and quantification is *Pfs25* mRNA [9], which is gametocyte-specific and highly conserved among different parasite isolates [6]. Although expression of *Pfs25* mRNA is upregulated in female gametocytes [10], the female-biased gametocyte sex ratio and the high abundance of *Pfs25* mRNA relative to the male specific marker *Pfs230p* make it an attractive target for sensitive gametocyte detection and quantification [11].

Various molecular techniques have been used for gametocyte detection and quantification. For *Pfs25* mRNA there are two commonly used protocols, one based on quantitative reverse transcriptase PCR (qRT-PCR) and another based on quantitative nucleic acid sequence based amplification (QT-NASBA) [6, 12]. Although previous studies on other malaria-specific targets suggest that the accuracy, precision and operational attractiveness may differ between assays [13], QT-NASBA and qRT-PCR, as currently routinely used, have never been directly compared for *Plasmodium falciparum* gametocyte quantification. Additionally, differences in *Pfs25* transcript levels between malaria parasite strains have been hypothesized but never directly examined [14].

Here, *Pfs25* mRNA QT-NASBA and qRT-PCR were compared for intra- and inter-assay variation using gametocytes from three different isolates [6, 12]. The association between gametocyte densities estimated by these two molecular methods in natural malaria infections and infectiousness quantified by membrane feeding assays was also determined.

## Methods

### Study design

In this study, both parasite culture-derived samples and samples from naturally infected individuals were used. For the estimation of intra-assay variation for both QT-NASBA and qRT-PCR, culture-derived mature *P. falciparum* gametocytes at known densities were assayed in triplicates on the same 96-well plate with the same reaction mixtures. For the inter-assay variation analysis, gametocyte dilution series were tested on separate plates and with separately prepared reaction mixtures. Samples collected from naturally infected individuals living in malaria-endemic regions were used to assess the correlation between gametocyte levels measured by microscopy, qRT-PCR and QT-NASBA and human infectiousness to mosquitoes, determined by mosquito feeding experiments.

### Gametocyte culture

*Plasmodium falciparum* gametocytes from three laboratory parasite strains (NF54, NF135, NF166) were cultured in shaker flasks, as previously described [15–17]. Briefly, asexual blood stage parasites were reseeded at 5% haematocrit (erythrocytes provided by healthy Dutch donors with blood type A) and 0.5% parasitaemia in culture medium containing RPMI 1640 with HEPES (5.94 g/L), hypoxanthine (0.05 g/L), 10% (v/v) pooled human sera (also blood type A) obtained from malaria-naive individuals, and 0.2% (w/v) sodium bicarbonate. Culture medium was automatically changed twice a day, and on day 4, 50 mM of *N*-acetylglucosamine was added in order to prevent the next generation of merozoites from infecting new erythrocytes. Gametocytes were allowed to mature until day 14.

### Gametocyte dilution series

On day 14, mature gametocytes were harvested and Percoll purified according to the protocol described in Kariuki et al. [18]; 63%, instead of 65%, Percoll gradient was used. Cultures and material used for purification were continuously kept at 37 °C to avoid gametocyte activation. Following Percoll purification, gametocytes were counted by a single reader using a haemocytometer and diluted in whole blood at densities of 10^3^, 10^2^, 10^1^, 10^0^, 10^−1^, and 10^−2^ or 5 × 10^3^, 5 × 10^2^, 5 × 10^1^, 5 × 10^0^, 5 × 10^−1^, and 5 × 10^−2^ gametocytes per µL. Fifty µL aliquots of each dilution were further diluted in 250 µL of RNAprotect Cell Reagent (Qiagen) when analysing standard curve triplicates and samples collected in Burkina Faso (see below) or in 450 µL of L6 buffer when analysing samples collected in Mali, and stored at −80 °C until further use [19].

### Samples from naturally infected individuals in clinical trials

Blood samples from patent gametocyte carriers were collected during clinical trials in Mali and Burkina Faso [20, 21]. In the study undertaken in Mali, male participants with at least 32 gametocytes per μL of blood based on gametocyte enumeration against 500 white blood cells (WBC), assuming 8000 WBCs/µL, were recruited. The trial in Burkina Faso had as an inclusion criterion the presence of at least one microscopically detectable gametocyte at screening slides, where 100 microscopic fields were examined. In both studies, blood samples were taken at multiple time-points, before and after treatment initiation, to assess gametocyte clearance. Samples [venous blood in EDTA (Mali) or heparin (Burkina Faso) vacutainer] taken before initiation of anti-malarials were used in the current study. In Burkina Faso, 50 µL aliquots of blood were transferred into 250 µL of RNAprotect Cell Reagent. In Mali, 100 µL of blood were transferred into 900 µL of L6 buffer. Both sets of samples were kept at −80 °C until shipment, although the samples collected in Burkina Faso may have been exposed to freeze–thaw due to power-cut of unknown duration during political unrest in November 2014. For both studies, samples were shipped in dry ice at controlled temperature (−70 to −80 °C) to Radboud University Medical Centre (Nijmegen, The Netherlands), where the laboratory work was performed.

For all samples, total nucleic acids (NA) were extracted using a MagNAPure LC automatic extractor (Total Nucleic Acid Isolation Kit–High Performance, Roche Applied Science) and eluted into 50 µL of elution buffer; while all of the 300 µL of samples collected in Burkina Faso were extracted, only half of the volume (500 µL of the 1 mL) of samples collected in Mali were used for total NA extraction.

### Mosquito membrane feeding experiments

All individuals whose samples were included in this analysis participated in mosquito feeding experiments: a small quantity of heparinized venous blood (400–500 μL) was offered to female *Anopheles gambiae* mosquitoes (180 mosquitoes in the Mali study and 60 in the Burkina Faso study) through a membrane [20–22]. Fully fed mosquitoes at the end of each experiment were selected, kept for one week on glucose at 27–29 °C to allow for parasite development, and dissected. In both studies, light microscopy was used to detect oocysts on the midgut wall, and their presence was confirmed by a second microscopist. The proportion of mosquitoes with at least one oocyst was used as outcome measure.

### *Pfs25* mRNA qRT-PCR

The use of reverse transcriptase PCR (RT-PCR) for the detection of *Pfs25* mRNA was first described by Babiker et al. [23]; recently it was further developed into a qRT-PCR [6]. Here, a protocol described by Schneider et al. was used [11] with primers designed by Wampfler et al. [6]. Briefly, total NA extraction was followed by RQ1 DNaseI digest (Promega) and cDNA synthesis (High Capacity cDNA Reverse Transcription Kit, Applied Biosystems). The DNase treatment and the RT-step were done once for each sample; the qPCR step was done in triplicate for samples included in the intra-assay variation analysis. Success of the DNase treatment was tested by performing qPCR, with the same primers, on two to five randomly selected samples of each plate (in total 35 samples) before proceeding to the RT-step. No residual genomic DNA was detected in these assays, which indicates successful DNase treatment. PCR conditions were according to manufacturer’s instructions, described as standard cycling programme of GoTaq® qPCR Master Mix (Promega). qRT-PCR cycle of threshold (Ct) values (the cycle at which the fluorescence signal crosses a pre-determined threshold during the exponential phase of the amplification) were obtained using CFX96™ Real-Time PCR Detection System (BIO-RAD). For samples from naturally infected individuals, Ct values were converted into gametocyte density using plate-specific standard curve. Samples with estimated densities below 0.02 gametocytes per µL or 1 gametocyte per 50 µL of sample were considered negative. At the highest density of the gametocyte dilution series, cDNA corresponding to 363.6 gametocytes was estimated to have been included in qRT-PCR reactions (Additional file 1: Figure S1).

### *Pfs25* mRNA QT-NASBA

QT-NASBA was performed as in Schneider et al. [12]. KCl concentrations in experiments performed for this study varied from 50 to 60 mM, and differed from the concentration used in the original protocol (80 mM) [12]. For samples from the Burkina Faso study, the extracted total NA used for qRT-PCR was also used for QT-NASBA (before DNase treatment and cDNA production). For samples collected in the Mali trial, different aliquots of the same sample were extracted (total NA) and used for the different molecular assays; the QT-NASBA aliquot did not undergo DNAse treatment and cDNA production. QT-NASBA results are expressed as time to positivity (TTP), as in this method amplification happens at a constant temperature, not in cycles. Standardized manual thresholds to remove background noise are set at the begin value of 3 min and the end value of 15 min. As with qRT-PCR, gametocyte densities were assigned based on plate-specific gametocyte dilution series, which was diluted in whole blood before extraction of total NA. For samples from infected individuals, estimated gametocyte densities below 0.02 gametocytes per µL were considered to be negative. At the highest density of the gametocyte dilution curve, total NA corresponding to 2500 gametocytes was estimated to have been included in QT-NASBA reactions (Additional file 1: Figure S1).

### Statistical analysis

Analyses were conducted using STATA version 12 (Stata Corporation, College Station, TX, USA) and R (R Foundation for Statistical Computing, Vienna, Austria).

Intra-assay variation was assessed by estimating coefficients of variation (CV, expressed in %) (i.e., standard deviation divided by mean, and multiplied by 100) for each level of gametocytaemia tested in triplicate, in culture-specific gametocyte dilution series. Gametocyte dilution series were also used to quantify inter-assay variation: CV was estimated for each gametocyte density for all series tested in different reaction plates. To analyse the agreement between qRT-PCR- and QT-NASBA-defined gametocyte levels in samples from infected individuals in the Mali study, a Bland–Altman plot was constructed, whereby differences between densities assigned by these two methods are presented by the average assigned level. Generalized linear mixed models, with study participant as random effect, were fit to assess the association between mosquito infection status in feeding assays and gametocyte densities (fixed effect) assigned by these two molecular methods. For each quantification method, Akaike information criterion was used to select gametocyte density scale (linear or logarithmic) in the models. Marginal R^2^, variance explained by fixed effects, was estimated using the ‘r.squaredGLMM’ function in MuMIn package in R [24].

## Results

### Intra-assay variation

*Plasmodium falciparum* gametocytes of the NF54, NF135 and NF166 strains were used to prepare dilution series with 10^3^, 10^2^, 10^1^,10^0^, 10^−1^, and 10^−2^ gametocytes per μL. Additionally, for the NF54 strain, samples originating from three different culture flasks were tested. For each gametocyte density of individual dilution series, *Pfs25* mRNA expression was analysed in triplicate, using both qRT-PCR and QT-NASBA.

**Fig. 1:**
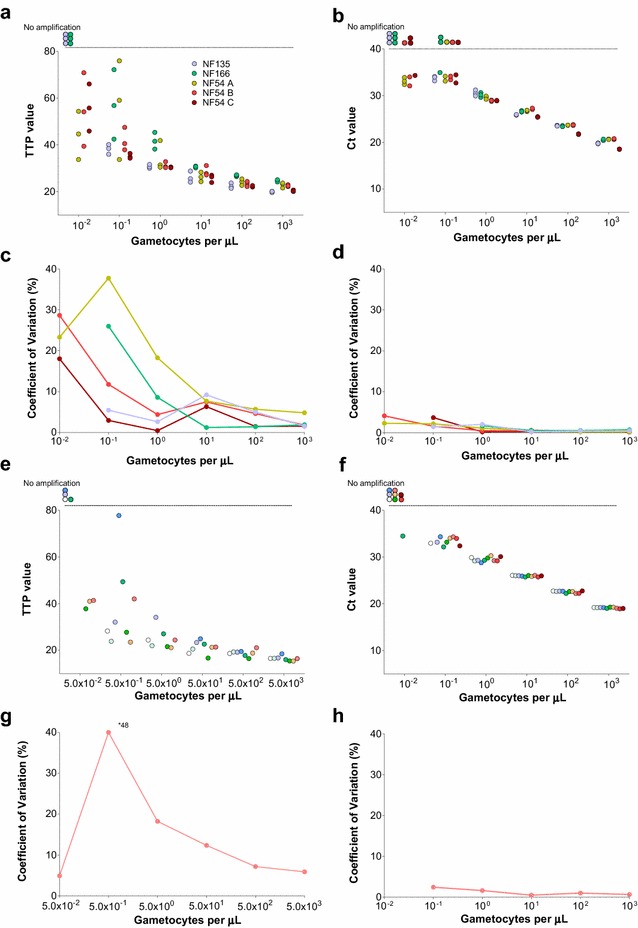
Intra (**a**–**d**) and inter (**e**–**h**) assay variation of QT-NASBA and qRT-PCR. In **a**–**d**, *different colours* represent different strains and cultures used for intra-assay variation assessment. In **e**–**h**, different experiments used in the inter-assay variation assessment are represented by *different colours*. Time to positivity (TTP) values for samples tested by QT-NASBA are presented in **a** and **e** and coefficients of variation (CVs), in **c** and **g**. Cycle of threshold (Ct) values for samples tested by qRT-PCR are presented in **b** and **f**; CVs are presented in **d** and **h**. In **a**, **b**, **e** and **f**, *circles* outside the *y-axis* range correspond to samples where *Pfs25* mRNA was not detected. Of note, for experiments included in inter-assay variation analysis, different densities were used in gametocyte dilution series for QT-NASBA and qRT-PCR

In Fig. 1 (panels a, b), TTP and Ct values for QT-NASBA and qRT-PCR, respectively, are presented. For both assays, *Pfs25* mRNA was not detected at the lowest gametocyte densities (0.01 gametocytes per µL) of the NF135 and NF166 dilution series. Although results from dilution series appeared comparable between all three *P. falciparum* strains, the failure to detect the lowest gametocyte concentration for NF135 and NF166 might indicate lower *Pfs25* transcript levels compared to the NF54 strain that only affects detection and quantification at extremely low densities. Additionally, in 5/15 0.10-gametocytes-per-µL samples, *Pfs25* mRNA was not detected by qRT-PCR. In samples where amplified *Pfs25* transcript was detected CV were inversely related to gametocyte levels and much lower for qRT-PCR compared to QT-NASBA at all gametocyte densities.

### Inter-assay variation

Seven (including the following densities 5 × 10^3^, 5 × 10^2^, 5 × 10^1^, 5 × 10^0^, 5 × 10^−1^, and 5 × 10^−2^ gametocytes per μL) and ten (including the densities 10^3^, 10^2^, 10^1^,10^0^, 10^−1^, and 10^−2^ gametocytes per μL) gametocyte dilution series, each tested on a different plate, were used to assess QT-NASBA and qRT-PCR inter-assay variation, respectively. Only NF54 strain parasite cultures were used in these experiments. Samples with the lowest density in each series were often negative for *Pfs25* mRNA. Despite the higher densities used for the QT-NASBA *versus* qRT-PCR dilution series, a similar pattern to the intra-assay variation experiments was observed (Fig. 1, panels g, h): CV were considerably higher for QT-NASBA compared to qRT-PCR and decreased with increasing gametocyte densities.

### Gametocyte densities in naturally infected individuals

**Fig. 2:**
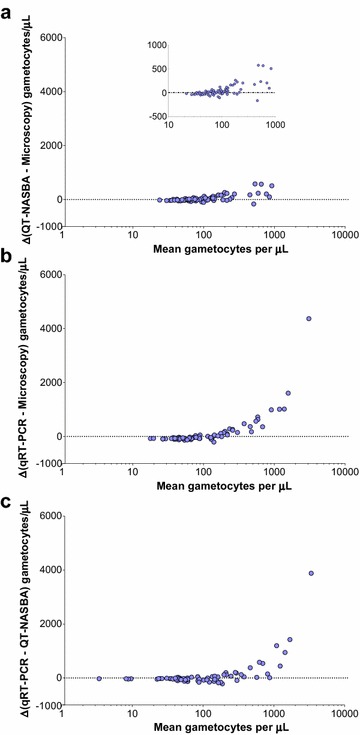
Bland-Altman plots to assess agreement between light microscopy, QT-NASBA and qRT-PCR levels in samples from naturally infected individuals. In the y-axes, differences between gametocyte levels estimated by two different methods are presented: **a** QT-NASBA levels minus microscopy-defined densities, **b** qRT-PCR minus microscopy levels, **c** qRT-PCR levels minus levels estimated by QT-NASBA. The *X-axes* present the average of the densities estimated by the two methods included in the calculation of the respective *Y-axes*. In panel **a**, the differences (*Y-axis*) are limited to a narrower range of values compared to panels **b** and **c**, and the inset plot presents the same data using different *Y-axis* limits

Blood samples collected prior to treatment from gametocytaemic individuals participating in mosquito feeding experiments in Mali were analysed by microscopy, qRT-PCR and QT-NASBA. For the two molecular assays, gametocyte densities were estimated based on dilution series of culture-derived *P. falciparum* gametocytes of the NF54 strain tested in the same plate, as routinely done [5, 12]. As a consequence of enrolment criteria, all individuals (N = 73) had microscopically detectable gametocytes at the time of the feeding assay (median (IQR) 80 (64–112) gametocytes per μL), and all their samples were assigned gametocyte levels above the threshold of positivity for both molecular assays (minima gametocyte levels were 0.04 and 6.1 gametocytes/µL for qRT-PCR and QT-NASBA, respectively). Median (IQR) gametocytaemias estimated by QT-NASBA and qRT-PCR were similar: 94.8 (41.6–180.7) and 69.8 (29.8–263.0) gametocytes per μL, respectively. The agreement between microscopy and QT-NASBA, between microscopy and qRT-PCR and between the two molecular methods was assessed by Bland–Altman plots (Fig. 2). At high gametocyte concentrations (>100 gametocytes per μL), gametocyte densities estimated by qRT-PCR were higher than densities estimated by microscopy or QT-NASBA.

**Fig. 3:**
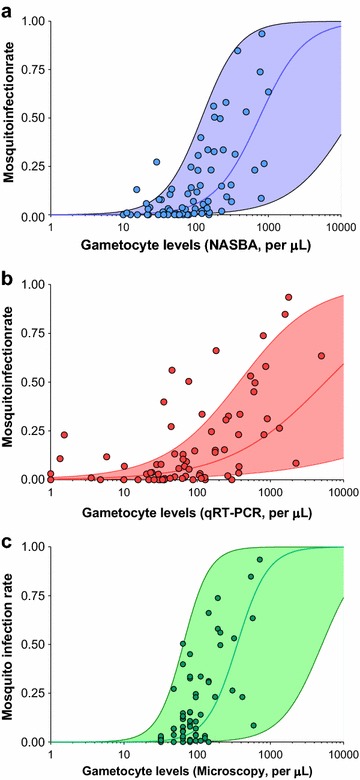
Mosquito infection rates (*y-axes*), presented as proportions, by gametocyte counts (*x-axes*) in samples collected in a clinical study in Mali. Gametocyte densities were estimated by QT-NASBA (**a**), qRT-PCR (**b**) and light microscopy (**c**). Fitted curves and 95% confidence intervals were estimated using glmmadmb package in R

In the same study in Mali, 73 membrane feeding assays were performed. The median (IQR) number of mosquitoes fed and dissected was 176 (174–177) and 142 (130–152), respectively. Overall 58/73 (79.4%) individuals infected at least one mosquito, and the median (IQR) mosquito infection rate (proportion of infected mosquitoes, i.e., proportion of mosquitoes with ≥ one oocyst) was 12.4% (3.4–32.6). Gametocyte levels were lower in non-infectious *versus* infectious individuals (Additional file 2: Figure S2), and correlated with mosquito infection rates (Spearman’s rank correlation coefficient 0.59, 0.62 and 0.64 (P < 0.001 for all assays) for qRT-PCR, microscopy and QT-NASBA, respectively). Statistical models including log-transformed gametocyte levels estimated by QT-NASBA or microscopy explained a slightly higher proportion (marginal R^2^ = 0.28 and 0.26, respectively) of infectiousness variability compared to models including log-transformed densities by qRT-PCR (marginal R^2^ = 0.22) (Fig. 3).

### Lower-than-expected gametocyte densities in molecular assays

Venous samples collected from 77/79 children participating in mosquito feeding experiments in Burkina Faso were also analysed. Of these 77 children, 27 did not carry microscopically detectable gametocyte densities on the day of their membrane feeding assay, despite having gametocytes detected by microscopy during the screening phase of the study (one to three days earlier). Median (IQR) gametocyte density determined by microscopy was 12 (0–24) gametocytes per μL. Twenty of 77 did not have *Pfs25* mRNA detected by qRT-PCR, and four had assigned gametocyte levels between 0 and 0.02 gametocytes per μL, and were considered gametocyte negative. However, ten of these 24 children with no gametocytes detected by qRT-PCR had gametocytes detected by microscopy. Gametocyte densities estimated by QT-NASBA were also very low for most samples: 55 children, including 31 with microscopically detectable gametocytes, had assigned levels below 0.02 gametocytes per μL (and were thus classified as gametocyte negative) and for one child of 76 *Pfs25* mRNA was not detected. All 23 samples with no *Pfs25* mRNA amplification or with densities below 0.02 gametocytes per μL by qRT-PCR and that were also tested by QT-NASBA had assigned densities below 0.02 gametocytes per μL by this method. Considering that all these children had microscopically detectable gametocytes one to three days before sample collection, it is likely that RNA material was of insufficient quality. It is highly plausible that freeze–thaws with unknown duration or frequency have affected RNA integrity of this set of samples, all kept in the same box throughout the storage period and shipment. The infectivity of study participants in the clinical trial in Burkina Faso was lower than the infectivity of individuals enrolled in the Malian study: 29/77 (37.6%) children infected at least one mosquito in feeding experiments. Although differences in the prevalence of host factors that influence infectivity, such as transmission-blocking immunity [25] or haemoglobinopathies [26], could have contributed to this observation, this disparity in infectiousness prevalence is probably due to real differences in gametocyte levels as suggested by microscopy-based quantification and differences in the number of dissected mosquitoes (median number of mosquitoes dissected per assay, 142 and 45 in Mali and Burkina Faso, respectively). The facts that five of 29 and 17 of 29 individuals who infected mosquitoes did not have *Pfs25* mRNA detected by qRT-PCR or had very low assigned levels (<0.02 gametocytes per μL) by QT-NASBA, respectively, and that the gametocyte density-mosquito infection rate plot is shifted to the left compared to the corresponding graph for the Malian study (Additional file 3: Figure S3) support the freeze–thaw explanation.

## Discussion

In this study, *Pfs25* mRNA QT-NASBA and qRT-PCR were evaluated using culture-derived gametocyte samples and samples from naturally infected individuals. In in vitro experiments, both intra- and inter-assay variations were lower for qRT-PCR compared to QT-NASBA. When analysing samples from naturally infected individuals, gametocyte densities estimated by qRT-PCR were higher than densities estimated by QT-NASBA and microscopy in individuals with high gametocyte levels. Gametocyte densities estimated by the two molecular assays, as well as by microscopy, correlated well with mosquito infection rates. Overall, the findings indicate that both assays are useful for quantifying sexual stage parasite densities in samples from naturally infected individuals, but qRT-PCR showed better reproducibility.

Determining the variability of quantitative assays is important for the interpretation of results. The experiments here suggest that qRT-PCR has lower intra- and inter-assay variation compared to QT-NASBA. The higher precision of qRT-PCR is evident for the entire range of gametocyte densities used in the dilution series, despite involving extra reaction steps, such as DNase treatment and cDNA production, which are known to be a source of variation [27]. These two steps were performed once for each dilution series density, for the three different NF54 cultures and the NF135 and the NF166 cultures. There was considerably less between-strain and -culture variation for similar densities in qRT-PCR *versus* QT-NASBA. In QT-NASBA reactions, from the time of primer depletion, amplification will mostly depend on the initial number of transcripts, resulting in a linear increase of amplicons. This exact moment when amplification starts may differ between samples depending on when and how the enzyme is added into the reaction wells, which adds a level of uncertainty to this methodology [28]. The range of TTP values in dilution series is also sensitive to the KCl concentration in the mix, which requires regular optimization with new targets, batches of primers or molecular beacons [29].

Despite qRT-PCR being a more precise method for gametocyte quantification, QT-NASBA might detect a larger number of very low gametocyte densities (<0.1 gametocytes per μL). QT-NASBA was originally developed for highly sensitive qualitative detection. The apparent higher sensitivity of QT-NASBA compared to qRT-PCR may be explained by methodological differences: for QT-NASBA it is not necessary to dilute samples, in order to complete DNase treatment and cDNA production (Additional file 1: Figure S1) and the equivalent of a larger volume of the original sample is therefore added into the final reaction in QT-NASBA compared to qRT-PCR. This additional dilution in the qRT-PCR protocol could be avoided by using column-based RNA extraction with on-column DNase digestion [6]. Also, while qRT-PCR, in the protocol used here, carries on for a set amount of 40 cycles, the QT-NASBA reaction is continuous at a stable temperature for the duration of 90 min. This might allow for more sensitive amplification of low numbers of transcripts, but could also lead to the detection of single stranded DNA, in cases in which genomic DNA has been degraded and exists in the sample in a single stranded form. A stringent cut-off density below which samples are considered negative must therefore be applied in QT-NASBA, as with all other molecular assays.

Whether qRT-PCR overestimates true gametocyte levels or QT-NASBA and microscopy underestimate densities is not clear. When qRT-PCR is compared with the other methods, high mean densities correspond to the qRT-PCR measurement being larger than the other measurements. There are two possible reasons for observing this pattern: (1) qRT-PCR overestimates high gametocyte concentrations; or, (2) qRT-PCR measurements have greater variability [30]. Since the in vitro experiments suggest that qRT-PCR has lower variability than QT-NASBA, it is likely that qRT-PCR has an upward bias at high gametocyte concentrations (>100 gametocytes per μL). The overestimation is unlikely to be caused by residual DNA in the RNA samples used in the qRT-PCR analysis, as the success of the DNase treatment was tested for two to five samples per plate, with no amplification detected.

The value of molecular methods in detecting epidemiologically relevant malaria infections is becoming increasingly well established. A recent meta-analysis comparing PCR, microscopy and rapid diagnostic test (RDT) sensitivities showed that on average RDTs detect less than half of PCR-positive *P. falciparum* infections, and microscopy detects slightly less infections than RDTs [8]. The density of these sub-microscopic infections and their concurrent gametocyte levels are of relevance in estimating the contribution to malaria transmission. For *P. falciparum* parasite density (including asexual parasites and gametocytes), RNA-based QT-NASBA was previously compared to DNA-based qPCR for the quantification of *P. falciparum 18S* RNA and DNA, respectively [13]. Another recent study, this time on *Leishmania* parasites, compared *18S* rRNA-based quantification by qRT-PCR and QT-NASBA to *18S* rRNA gene DNA-based detection with qPCR [31]. In the first study, QT-NASBA was found to be more convenient and equally applicable for quantification purposes as real-time PCR, with strong correlation with quantification by microscopy and similar inter-assay variation to both methods [13]. In the second study, intra- and inter-assay CV were deemed equal for all three molecular methods and qRT-PCR was preferred over the other two methods for convenience reasons [31]. One explanation for these conflicting conclusions is the method used for detection of the amplified target in QT-NASBA: in the study comparing *Leishmania* quantification, the electrochemiluminescence (ECL) read-out added to the general workload of the assay. Additionally, co-amplification with a known amount of quantitative (Q)-RNA, as was originally done for the quantification of the different gametocyte stages as well as the *Leishmania* parasites, added an extra factor to the analysis of results [12, 31]. The direct comparison to a standard curve with microscopically determined density of parasites, in addition to the use of molecular beacons, simplified the quantification process of QT-NASBA results [13]. As mentioned earlier in the context of assay sensitivity, also convenience-wise, an important advantage of QT-NASBA is that it can be performed directly on extracted NA.

Several factors, such as the type of anticoagulant used in sample collection and the storage conditions, might influence the outcome of molecular assays on samples [9, 32]. The analysis of samples from Burkina Faso provides indirect evidence for this: the extremely low assigned gametocyte levels, including in samples collected from children with microscopically detectable gametocytes, suggest that these samples might have gone through more than one freeze-thaw cycle. In these situations, sample quality may be compromised: mRNA may be degraded and, although abundant transcripts, such as *Pfs25* mRNA, might still be detectable, quantification becomes less reliable [33]. Quantification of constitutively expressed human RNA targets and comparison of transcript levels among samples is one method that could have confirmed whether RNA degradation occurred and is recommended for future studies where there is uncertainty about sample integrity.

Ultimately, the goal in quantifying gametocytes is to indirectly estimate human infectivity, as mosquito feeding assays are logistically complex and only a handful of research institutes currently have the infrastructure to perform these experiments in sub-Saharan Africa. In this study, it was observed that QT-NASBA- and microscopy-defined densities explain a slightly higher proportion of the variation in infectivity compared to gametocyte levels assigned by qRT-PCR but this difference should be interpreted with caution. The statistical method used here to evaluate this relationship was recently employed to assess the association between viraemia and mosquito infection risk in feeding assays involving dengue-infected individuals [34]. While the sigmoidal curves estimated by these models seem to fit mosquito infection data well for QT-NASBA and microscopy, it is possible that qRT-PCR-defined high densities were overestimated (Fig. 2b) and that could possibly explain the poorer fit of the model for that assay. These comparisons are based on a statistical model. More complex models [35], allowing for more flexible sigmoidal curves, would be required to formally assess the shape of the association between gametocyte density and mosquito infection rates. The quantification of other parasite (e.g., sex ratio) or host factors (e.g., transmission-blocking immune responses and haematological factors) that might influence infectivity is likely to improve the predictive value of these models [36]. Of note, the enrolment criterion of multiple gametocytes being observed by microscopy (two or more gametocytes per 500 WBC) makes it difficult to directly extrapolate these results to sub-microscopic infections. By definition, microscopy has no quantitative value in sub-patent infections and molecular methods will need to be used to characterize the gametocytaemia-infectivity curve at low gametocyte densities.

Despite being the most commonly used amplification target for estimating mature *P. falciparum* gametocytes counts, the use of *Pfs25* transcripts for quantifying gametocyte densities has drawbacks. *Pfs25* mRNA levels are much lower in male compared to female gametocytes [11]. Although it is generally assumed, due to the female bias in sex ratios (4–5:1) in natural infections, that female gametocyte levels are a good surrogate for all-sex gametocytes counts, quantification of the less abundant male gametocyte-specific *Pfs230p* mRNA or other male targets would provide more accurate estimates of the total number of mature gametocytes as well as sex ratios [11], a factor known to influence infectiousness [37]. In addition to these targets, transcribed only in mature gametocytes, transcripts of other genes have been used for the study of sexual stage malaria: an alternative marker of female gametocytes is *pfg377*, the expression of which begins in the sequestered stage III [38]; a traditional marker for commitment to gametocytogenesis is *Pfs16* [12, 39], which is present in all gametocyte stages. Another early marker of commitment is *PfGEXP5*, the expression of which starts in ring-stage parasites committed to gametocytogenesis, is a potentially useful tool to be used in studies aiming to identify factors influencing commitment to sexual stage [40]. For all of these targets, transcript detection is possible both by the more convenient QT-NASBA and by the more laborious but more reproducible qRT-PCR. Design of intron-spanning primers may close the gap between the attractiveness of both methods and retain the advantage of the more reproducible qRT-PCR [41].

## Conclusion

Estimating the contribution of sub-microscopic infections to malaria transmission is a priority in the malaria elimination era. Molecular methods that can quantify gametocytes levels in microscopically undetectable infections come with a promise to improve understanding of malaria epidemiology [42]. qRT-PCR of *Pfs25* mRNA is more reproducible compared to QT-NASBA. Gametocyte densities estimated by both methods, and by microscopy, correlate well with infectiousness in untreated individuals. Although this analysis of field samples suggest that these assays are suitable to quantify patent gametocyte levels, their use to understand the detectability and infectivity of sub-microscopic gametocyte densities is even more important. Future studies thus need to be designed to include individuals with sub-patent gametocyte levels to confirm the sensitivity, precision and relative merits of these assays. Precise quantification of gametocytes at very low levels is of relevance to determine if a threshold density is associated with infectivity provided molecular targets are informative of gametocyte viability and not merely of density [43]. Importantly, neither of the two molecular methods is routinely used in areas where field studies are undertaken and both assays are highly dependent on sample RNA quality.

## Additional files

**Additional file 1: Figure S1.** Estimated gametocyte nucleic acid quantities in each reaction step based on highest dilution of gametocyte standard curve (10^3^ gametocytes/μL). For dilution series used to assign gametocyte levels to field samples, the RNA stabilising buffer was L6 Buffer (450 μL) for the Mali study and RNAprotect (250 μL) for the Burkina Faso study.

**Additional file 2: Figure S2.** Gametocyte levels by infectiousness. Individuals who infected less than the median proportion of infected mosquitoes in positive assays (12.4%) were considered to have low infectiousness. Three samples with qRT-PCR levels lower than 1 gametocyte/μL had their values set to 1 gametocyte/μL so they could be represented in this graph.

**Additional file 3: Figure S3.** Gametocyte levels and mosquito infection rates in the Burkina Faso (A) and Mali (B) studies. Individuals who infected at least one mosquito are presented in this graph. In (A), data from the Burkina Faso transmission study is presented; light blue circles with donut hole represent samples with assigned gametocyte levels by QT-NASBA below 0.02 gametocytes/μL. Red circles with donut hole represent 5 samples for which the *Pfs25* amplicon was not detected by qRT-PCR; in this graph, these qRT-PCR negative samples were assigned the value of 0.02 gametocytes/μL. One sample had levels quantified by qRT-PCR below 0.02 gametocytes/μL. In (B), similar data from the Mali study is presented using the same x-axis range for comparison; black circles represent gametocyte levels estimated by microscopy.
