# Supplementary material for: Comparison of molecular quantification of Plasmodium falciparum gametocytes by Pfs25 qRT-PCR and QT-NASBA in relation to mosquito infectivity
Source: Malar J. 2016 Nov 8;15:539. doi: 10.1186/s12936-016-1584-z (PMC5100312; doi:10.1186/s12936-016-1584-z)
Supplement: Supplementary file 1 — Additional file 1: Figure S1. Estimated gametocyte nucleic acid quantities in each reaction step based on highest dilution of gametocyte standard curve (103 gametocytes/μL). For dilution series used to assign gametocyte levels to field samples, the RNA stabilising buffer was L6 Buffer (450 μL) for the Mali study and RNAprotect (250 μL) for the Burkina Faso study. [file 12936_2016_1584_MOESM1_ESM.docx]

**Fig. S1** Estimated gametocyte nucleic acid quantities in each reaction step based on highest dilution of gametocyte standard curve (10^3^ gametocytes/μL). For dilution series used to assign gametocyte levels to field samples, the RNA stabilising buffer was L6 Buffer (450 μL) for the Mali study and RNAprotect (250 μL) for the Burkina Faso study.
