# Supplementary material for: Comparison of molecular quantification of Plasmodium falciparum gametocytes by Pfs25 qRT-PCR and QT-NASBA in relation to mosquito infectivity
Source: Malar J. 2016 Nov 8;15:539. doi: 10.1186/s12936-016-1584-z (PMC5100312; doi:10.1186/s12936-016-1584-z)
Supplement: Supplementary file 2 — Additional file 2: Figure S2. Gametocyte levels by infectiousness. Individuals who infected less than the median proportion of infected mosquitoes in positive assays (12.4%) were considered to have low infectiousness. Three samples with qRT-PCR levels lower than 1 gametocyte/μL had their values set to 1 gametocyte/μL so they could be represented in this graph. [file 12936_2016_1584_MOESM2_ESM.docx]

**Fig. S2** Gametocyte levels by infectiousness. Individuals who infected less than the median proportion of infected mosquitoes in positive assays (12.4%) were considered to have low infectiousness. Three samples with qRT-PCR levels lower than 1 gametocyte/μL had their values set to 1 gametocyte/μL so they could be represented in this graph.
