# Supplementary material for: Comparison of molecular quantification of Plasmodium falciparum gametocytes by Pfs25 qRT-PCR and QT-NASBA in relation to mosquito infectivity
Source: Malar J. 2016 Nov 8;15:539. doi: 10.1186/s12936-016-1584-z (PMC5100312; doi:10.1186/s12936-016-1584-z)
Supplement: Supplementary file 3 — Additional file 3: Figure S3. Gametocyte levels and mosquito infection rates in the Burkina Faso (A) and Mali (B) studies. Individuals who infected at least one mosquito are presented in this graph. In (A), data from the Burkina Faso transmission study is presented; light blue circles with donut hole represent samples with assigned gametocyte levels by QT-NASBA below 0.02 gametocytes/μL. Red circles with donut hole represent 5 samples for which the Pfs25 amplicon was not detected by qRT-PCR; in this graph, these qRT-PCR negative samples were assigned the value of 0.02 gametocytes/μL. One sample had levels quantified by qRT-PCR below 0.02 gametocytes/μL. In (B), similar data from the Mali study is presented using the same x-axis range for comparison; black circles represent gametocyte levels estimated by microscopy. [file 12936_2016_1584_MOESM3_ESM.docx]

**Fig. S3** Gametocyte levels and mosquito infection rates in the Burkina Faso (A) and Mali (B) studies. Individuals who infected at least one mosquito are presented in this graph. In (A), data from the Burkina Faso transmission study is presented; light blue circles with donut hole represent samples with assigned gametocyte levels by QT-NASBA below 0.02 gametocytes/μL. Red circles with donut hole represent 5 samples for which the *Pfs25* amplicon was not detected by qRT-PCR; in this graph, these qRT-PCR negative samples were assigned the value of 0.02 gametocytes/μL. One sample had levels quantified by qRT-PCR below 0.02 gametocytes/μL. In (B), similar data from the Mali study is presented using the same x-axis range for comparison; black circles represent gametocyte levels estimated by microscopy.
